# A radiomics-based model to classify the etiology of liver cirrhosis using gadoxetic acid-enhanced MRI

**DOI:** 10.1038/s41598-021-90257-9

**Published:** 2021-05-24

**Authors:** Aboelyazid Elkilany, Uli Fehrenbach, Timo Alexander Auer, Tobias Müller, Wenzel Schöning, Bernd Hamm, Dominik Geisel

**Affiliations:** 1 Department of Diagnostic and Interventional Radiology, Charité-Universitätsmedizin Berlin, Corporate Member of Freie Universität Berlin, Humboldt-Universität Zu Berlin, Berlin Institute of Health, Augustenburger Platz 1, 13353 Berlin, Germany; 2 Berlin Institute of Health (BIH), Anna-Louisa-Karsch-Straße 2, 10178 Berlin, Germany; 3 Division of Gastroenterology and Hepatology, Department of Medicine, Charité-Universitätsmedizin Berlin, Corporate Member of Freie Universität Berlin, Humboldt-Universität Zu Berlin, Berlin Institute of Health, Augustenburger Platz 1, 13353 Berlin, Germany; 4 Department of General, Visceral and Transplantation Surgery, Charité-Universitätsmedizin Berlin, Corporate Member of Freie Universität Berlin, Humboldt-Universität Zu Berlin, Berlin Institute of Health, Augustenburger Platz 1, 13353 Berlin, Germany

**Keywords:** Hepatology, Liver diseases, Machine learning

## Abstract

The implementation of radiomics in radiology is gaining interest due to its wide range of applications. To develop a radiomics-based model for classifying the etiology of liver cirrhosis using gadoxetic acid-enhanced MRI, 248 patients with a known etiology of liver cirrhosis who underwent 306 gadoxetic acid-enhanced MRI examinations were included in the analysis. MRI examinations were classified into 6 groups according to the etiology of liver cirrhosis: alcoholic cirrhosis, viral hepatitis, cholestatic liver disease, nonalcoholic steatohepatitis (NASH), autoimmune hepatitis, and other. MRI examinations were randomized into training and testing subsets. Radiomics features were extracted from regions of interest segmented in the hepatobiliary phase images. The fivefold cross-validated models (2-dimensional—(2D) and 3-dimensional—(3D) based) differentiating cholestatic cirrhosis from noncholestatic etiologies had the best accuracy (87.5%, 85.6%), sensitivity (97.6%, 95.6%), predictive value (0.883, 0.877), and area under curve (AUC) (0.960, 0.910). The AUC was larger in the 2D-model for viral hepatitis, cholestatic cirrhosis, and NASH-associated cirrhosis (P-value of 0.05, 0.05, 0.87, respectively). In alcoholic cirrhosis, the AUC for the 3D model was larger (P = 0.01). The overall intra-class correlation coefficient (ICC) estimates and their 95% confident intervals (CI) for all features combined was 0.68 (CI 0.56–0.87) for 2D and 0.71 (CI 0.61–0.93) for 3D measurements suggesting moderate reliability. Radiomics-based analysis of hepatobiliary phase images of gadoxetic acid-enhanced MRI may be a promising noninvasive method for identifying the etiology of liver cirrhosis with better performance of the 2D- compared with the 3D-generated models.

## Introduction

Liver cirrhosis—the end-stage of various types of chronic liver disease—is the 11th most common cause of death worldwide^1^. Liver transplantation is the only definitive treatment^1^. Patient management otherwise crucially relies on the screening for and management of serious complications such as hepatocellular carcinoma and gastroesophageal varices^2^. Chronic infection with hepatitis C virus and hepatitis B virus and alcoholic liver disease are the most common etiologies of liver cirrhosis worldwide^2,3^. Identification of the underlying etiology is important for treatment selection, alleviation of disease progression, and the allocation of transplant organs including posttransplant follow-up^4^.

Liver biopsy has been the reference standard for diagnosing the etiology of liver cirrhosis^5^. However, biopsy is invasive with an incidence of moderate to major procedure-related complications of approx. 1.2% and a mortality rate of 0.4%. Other limitations of liver biopsy include inter- and intraobserver variability and sampling error^6–8^. Such limitations emphasize the need for developing an alternative noninvasive method for identifying the etiology of liver cirrhosis especially in patients with indeterminate findings based on standard noninvasive diagnostic algorithms including physical examination, laboratory testing, biochemical markers, and imaging modalities^5,6,9,10^.

Gadoxetic acid-enhanced MRI provides morphological information on liver parenchyma, blood vessels, and the biliary tree (both anatomical and functional) while at the same time allowing detection and characterization of hepatic focal lesions as well as estimating functional liver capacity. Hepatocyte function—the main determinant of gadoxetic acid uptake and excretion—is known to be impaired in patients with liver cirrhosis^11–13^.

Radiomics analysis is a new technology based on the extraction of quantitative high-throughput features from radiologic images. The implementation of radiomics in radiology is gaining interest due to a wide range of applications such as its potential ability to characterize focal lesions including evaluation of tumor heterogeneity and microenvironment, phenotype classification, and prediction of response to treatment. In addition, radiomics models modified using the selected features can improve diagnostic accuracy, predict prognosis, and guide the clinical decision-making process^10,14–17^.

We hypothesize that a radiomics model based on features extracted from gadoxetic acid-enhanced MRI may allow identification and assessment of imaging features specific for different etiologies of liver cirrhosis and thus improve the classification of cirrhosis etiologies in patients with an inconclusive diagnosis based on currently available noninvasive diagnostic tests. Therefore, the purpose of our study was to develop, train, and validate a radiomics-based model as a noninvasive tool to predict the etiology of liver cirrhosis using features extracted from hepatobiliary phase (HBP) images of gadoxetic acid-enhanced MRI.

## Results

### Demographic data

The study included 248 patients (mean age, 60.5 ± 13.3 years; age range, 14–88 years), among them 179 men (mean age, 60 ± 12.8 years; age range, 14–81 years) and 69 women (mean age, 62 ± 14.3 years; age range, 21–88 years). Patient demographics are presented in Table 1.

**Table 1:** Summary of patient demographics.

Variable	n	Mean ± SD (min–max)
Female/male	69/179	–
Age at time of MRI acquisition (years)	248	60.5 ± 13.3 (14–88)
Amount of contrast medium (ml)	306	8.1 ± 1.4 (5–10)
APRI score	281	1.615 ± 0.079 (0.179–9.216)
Bilirubin (mg/dl)	287	1.7 ± 1.8 (0.18–11.1)
AST (U/L)	284	67.6 ± 39.3 (16–243)
ALT (U/L)	286	53.6 ± 46.5 (11–362)
GGT (U/L)	284	181.6 ± 185.9 (16–1068)
ALP (U/L)	282	159.5 ± 111.3 (35–869)
Albumin (gm/L)	161	3.5 ± 0.7 (2.03–5.2)
Platelets (× 10^9^/L)	284	141.4 ± 83.2 (26–467)
INR	283	1.25 ± 0.28 (0.8–2.9)
Creatinine (mg/dl)	287	0.89 ± .48 (0.4–5.9)
**Hepatic tumor**		
None	191	
Hepatocellular carcinoma	110	
Other malignancy (cholangiocarcinoma)	2	
Benign tumors^a^	3	
**Etiology of liver cirrhosis**		
Group 1: Alcoholic cirrhosis	108	
Group 2: Viral hepatitis-induced cirrhosis	93	
a. Hepatitis C virus (HCV)	71	
b. Hepatitis B virus (HBV)	15	
c. HBV-hepatitis D virus (HDV)	6	
d. HBV-HDV-HCV	1	
Group 3: Cholestatic liver disease	58	
a. Primary sclerosing cholangitis (PSC)	50	
b. Secondary biliary cirrhosis (SBC)	5	
Biliary atresia	2	
Caroli syndrome	1	
Congenital bile duct hypoplasia	1	
Recurrent pyogenic cholangitis	1	
c. Primary biliary cirrhosis (PBC)	2	
d. Secondary sclerosing cholangitis (SSC)	1	
Group 4: NASH-associated cirrhosis	28	
Group 5: AIH-associated cirrhosis	8	
Group 6: Other etiologies:	11	
a. Storage disease:	7	
1. Wilson disease	4	
2. Hemochromatosis type I	2	
3. Alpha-1 anti-trypsin deficiency	1	
b. Cystic fibrosis	2	
c. Budd-Chiari syndrome (BCS)	1	
d. Drug-induced (azathioprine)	1	
**Indications for gadoxetic acid-enhanced MRI**		
1. Workup of patients with liver cirrhosis	8	
2. Screening for suspected focal lesion	178	
3. Characterization of focal lesion	66	
4. Evaluation for liver transplantation	15	
5. Evaluation of patients with PSC	35	
6. Evaluation for TIPS	2	
7. Evaluation of jaundice	2	

*APRI score* AST-to-platelet ratio index, *AST* aspartate aminotransferase, *ALT* alanine aminotransferase, *GGT* gamma-glutamyl transferase, *ALP* alkaline phosphatase, *INR* international normalized ratio, *NASH* nonalcoholic steatohepatitis, *AIH* autoimmune hepatitis, *TIPS* transjugular intrahepatic portosystemic shunt.

^a^Focal nodular hyperplasia (n = 1), angiomyolipoma (n = 1).

### Differentiating between all included etiologies of liver cirrhosis

In one-vs-one multiclass classification differentiating between all 6 etiologies in the training subset, the fivefold cross-validated linear support vector machine (SVM) yielded the highest accuracy (52.8–82.5% for models derived from two-dimensional (2D) region of interest (ROI) and 53.6–78.5% models derived from three-dimensional (3D) volume of interest (VOI)). The highest sensitivity was noted with alcoholic cirrhosis (74.1% for 2D-ROI-derived models and 75.9% for 3D-VOI-derived models) and the highest specificity with cholestatic liver disease-induced cirrhosis (95% for 2D and 86.7 for 3D). Without validation, the fine K nearest neighbor (KNN) classifier had the highest accuracy of 100% (Table 2).

**Table 2:** Performance metrics of machine learning-based classification of radiomics features in the training subset.

	Linear support vector machine (SVM)							Subspace discrimination	
	Alcoholic cirrhosis		Viral hepatitis		Cholestatic liver disease			Cholestatic liver disease	
	2D	3D	2D	3D	2D		3D	2D	3D
Sensitivity	0.741	0.759	0.419	0.294	0.271		0.390	0.976	0.956
Specificity	0.412	0.414	0.836	0.832	0.950		0.867	0.458	0.441
Accuracy	0.528	0.536	0.725	0.690	0.825		0.785	0.876	0.856
Positive predictive value	0.406	0.414	0.482	0.386	0.552		0.377	0.883	0.877
Negative predictive value	0.746	0.759	0.799	0.766	0.852		0.873	0.818	0.703
False positive rate	0.588	0.586	0.164	0.168	0.050		0.133	0.542	0.559
False negative rate	0.259	0.241	0.581	0.706	0.729		0.610	0.024	0.045
False discovery rate	0.594	0.586	0.519	0.614	0.448		0.623	0.117	0.123
F1 score	0.525	0.536	0.448	0.333	0.364		0.383	0.927	0.915
MCC	0.152	0.173	0.267	0.138	0.299		0.253	0.551	0.479

*MCC* Matthews correlation coefficient.

### Differentiating cholestatic liver disease-induced cirrhosis from noncholestatic etiologies

**Figure 1:**
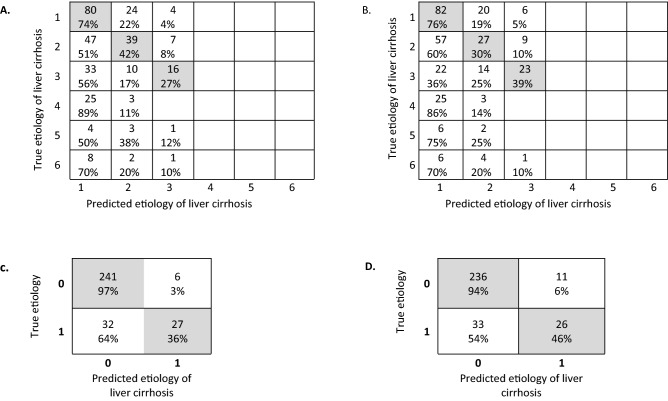
Confusion matrix of the training subset showing etiology predicted by the radiomics model in comparison to the diagnostically established etiology of liver cirrhosis. The shaded cells indicate correct predictions by the radiomics model. A and B are confusion matrices for all groups constructed using features extracted from 2-dimensional (2D) (**A**) and 3-dimensional (3D) (**B**) features. C and D are confusion matrices for noncholestatic (0) vs. cholestatic (1) liver cirrhosis in 2D (**C**) and 3D (**D**) models.

In binary classification differentiating cholestatic liver disease-induced cirrhosis (group 3) from noncholestatic etiologies of cirrhosis (groups 1, 2, 4–6) in the training subset, the fivefold cross-validated ensemble classifier—subspace discrimination—had the highest accuracy (87.6% and 85.6%), sensitivity (97.6% and 95.6%), positive predictive value (0.883 and 0.877), and the largest area under the curve (AUC) (0.83 and 0.80) in 2D- and 3D-derived models, respectively (Table 2). Confusion matrices are listed in Fig. 1.

In logistic regression analysis of the testing subset, three of the 45 features extracted and analyzed were omitted (Histo_Excess Kurtosis, Histo_Entropy_log2, GLCM_Entropy_log2) because of collinearity. The largest AUC was observed for differentiating cholestatic liver disease-induced cirrhosis from noncholestatic etiologies (AUC = 0.960 for 2D-derived models and 0.910 for 3D-derived models, P < 0.001).

### 2D- vs. 3D-generated radiomics models

**Figure 2:**
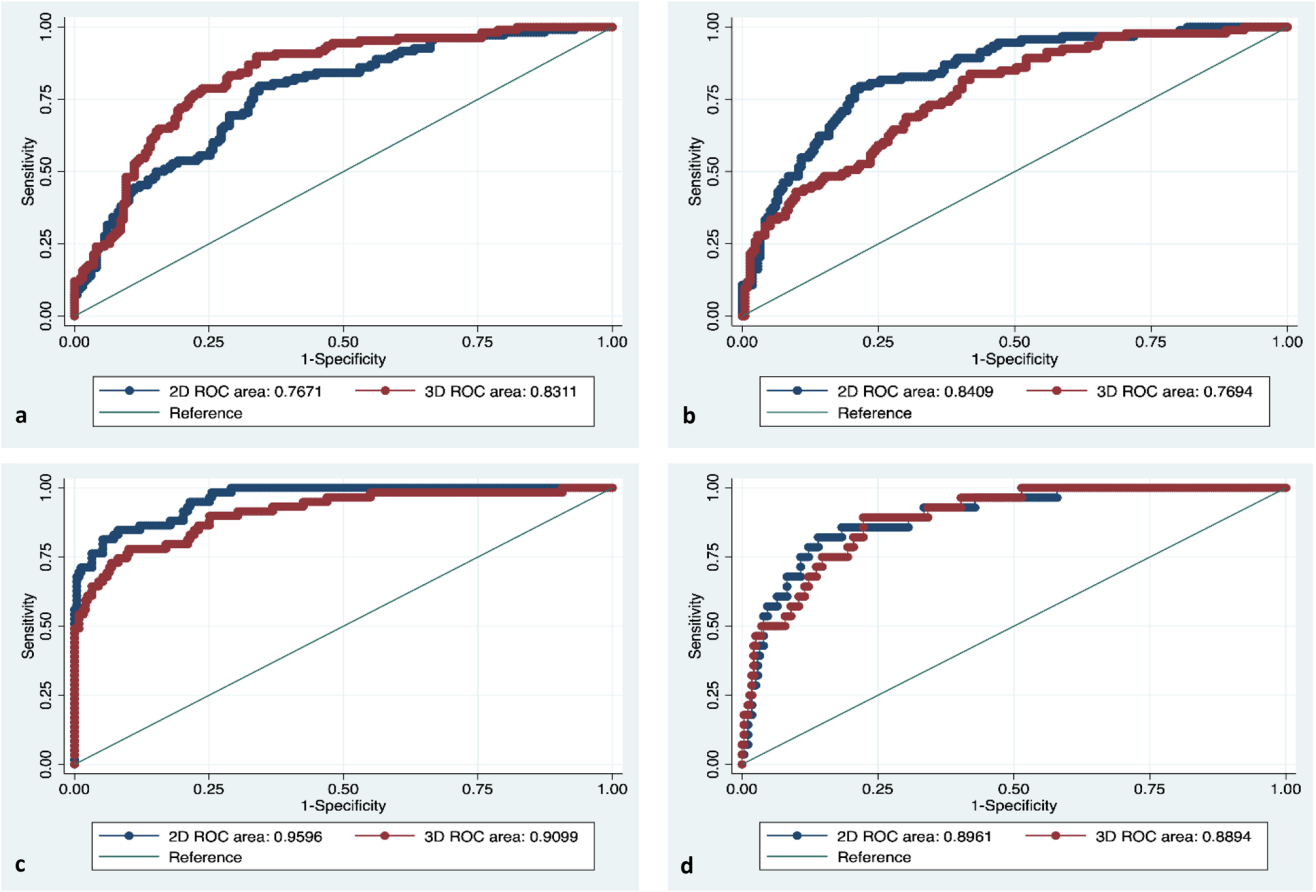
ROC curves of the testing subset for prediction of different etiologies of liver cirrhosis. Prediction of different etiologies of liver cirrhosis using one-vs-all multiclass logistic regression comparison between 2D- and 3D-extracted features in the following subgroups: alcoholic cirrhosis (**a**), viral hepatitis (**b**), cholestatic liver disease (**c**), and nonalcoholic steatohepatitis (NASH)-associated cirrhosis (**d**).

Comparison of radiomics models constructed using features extracted from 2D ROIs and 3D VOIs revealed larger AUCs in 2D-based models for viral hepatitis (P = 0.05), cholestatic liver disease (P = 0.05), and nonalcoholic steatohepatitis (NASH) (P = 0.87). In alcoholic cirrhosis (group 1), the model constructed using 3D features had a larger AUC (0.831 for 3D-VOI-derived models vs. 0.767 for 2D-ROI-derived models, P = 0.01) (Table 3, Fig. 2). The model differentiating cholestatic from noncholestatic liver cirrhosis had the largest number of statistically significant features (Table 4).

**Table 3:** Logistic regression analysis of the testing subset for alcoholic cirrhosis, viral hepatitis-induced cirrhosis, cholestatic liver disease-induced cirrhosis, and NASH-associated cirrhosis.

	ROI	N of features	ROC area	SE	[95% conf. interval]		LR chi^2^	P-value	Chi^2^	P-value
Alcoholic cirrhosis	2D	42	0.767	0.028	0.713	0.821	67.31	0.01	3.09	0.01
	3D	42	0.831	0.024	0.785	0.877	102.92	< 0.001		
Viral hepatitis	2D	42	0.841	0.024	0.794	0.887	102.46	< 0.001	3.75	0.05
	3D	42	0.769	0.028	0.714	0.825	64.95	0.01		
Cholestatic liver disease	2D	42	0.960	0.011	0.937	0.982	187.60	< 0.001	3.70	0.05
	3D	42	0.910	0.023	0.864	0.955	141.13	< 0.001		
NASH cirrhosis	2D	42	0.896	0.029	0.840	0.952	61.87	0.02	0.03	0.87
	3D	42	0.889	0.027	0.836	0.943	62.99	0.02		

*NASH* nonalcoholic steatohepatitis.

**Table 4:** List of statistically significant features in regression analysis for alcoholic cirrhosis, viral hepatitis-induced cirrhosis, cholestatic liver disease-induced cirrhosis, and NASH-associated cirrhosis (groups 1–4).

	Features	Coef.	SE	z	P > |z|	[95% conf. interval]	
**Alcoholic cirrhosis**							
2D	CONVENTIONAL_max	0.0712956	0.0348565	2.05	0.04	0.0029781	0.1396132
3D	CONVENTIONAL_max	0.1989438	0.075095	2.65	0.01	0.0517602	0.3461273
	GLRLM_LRE	− 1050.114	453.8196	− 2.31	0.02	− 1939.584	− 160.6442
	GLRLM_LRHGE	0.426994	0.2022717	2.11	0.04	0.0305487	0.8234393
	NGLDM_Coarseness	3051.282	1284.05	2.38	0.02	534.5903	5567.974
	NGLDM_Busyness	− 23.82545	11.01355	− 2.16	0.03	− 45.41161	− 2.239284
	GLZLM_LZE	0.1837164	0.0641918	2.86	0.004	0.0579028	0.3095301
	GLZLM_LZLGE	− 53.82372	21.954	− 2.45	0.01	− 96.85278	− 10.79467
	GLZLM_LZHGE	− 0.0000814	0.0000276	− 2.95	0.003	− 0.0001354	− 0.0000273
**Viral hepatitis-induced cirrhosis**							
2D	CONVENTIONAL_mean	1.670268	0.5492802	3.04	0.002	0.593699	2.746838
	CONVENTIONAL_max	− 0.1798322	0.0577049	− 3.12	0.002	− 0.2929318	− 0.0667326
	CONVENTIONAL_Q1	− 0.4539752	0.2094595	− 2.17	0.03	− 0.8645083	− 0.0434422
	CONVENTIONAL_Q2	− 0.5317651	0.1754611	− 3.03	0.002	− 0.8756625	− 0.1878677
	GLRLM_SRE	− 924.9035	381.044	− 2.43	0.02	− 1671.736	− 178.0709
	GLRLM_SRLGE	141,500.8	67,559.34	2.09	0.04	9086.91	273,914.7
3D	CONVENTIONAL_max	− 0.1075124	0.0513998	− 2.09	0.04	− 0.2082541	− 0.0067707
	GLZLM_ZLNU	0.0130915	0.0055781	2.35	0.02	0.0021587	0.0240244
**Cholestatic liver disease**							
2D	CONVENTIONAL_Q1	0.7107639	0.3471205	2.05	0.04	0.0304201	1.391108
	GLCM_HomoInver	− 791.7399	303.4408	− 2.61	0.01	− 1386.473	− 197.0068
	GLCM_ContrastVariance	2.333679	0.7833899	2.98	0.003	0.7982625	3.869095
	GLCM_Correlation	− 83.41012	31.7738	− 2.63	0.01	− 145.6856	− 21.13461
	GLCM_Entropy_log10	165.4433	57.34275	2.89	0.004	53.05358	277.833
	GLCM_Dissimilarity	− 75.27131	22.82484	− 3.30	0.001	− 120.0072	− 30.53545
	GLRLM_RP	− 2431.182	1162.547	− 2.09	0.04	− 4709.732	− 152.632
	NGLDM_Coarseness	− 5429.448	1713.633	− 3.17	0.002	− 8788.107	− 2070.79
	GLZLM_ZLNU	− 0.0189983	0.0081357	− 2.34	0.02	− 0.034944	− 0.0030526
3D	GLCM_Entropy_log10	134.0574	58.20325	2.30	0.02	19.98115	248.1337
	GLRLM_HGRE	3.580224	1.734314	2.06	0.04	0.1810302	6.979418
	GLRLM_SRHGE	− 2.977383	1.476149	− 2.02	0.04	− 5.870582	− 0.0841828
	GLRLM_LRHGE	− 0.5973161	0.2734658	− 2.18	0.03	− 1.133299	− 0.061333
	NGLDM_Coarseness	− 7320.082	3178.125	− 2.30	0.02	− 13,549.09	− 1091.071
	GLZLM_HGZE	− 0.0655245	0.033125	− 1.98	0.048	− 0.1304483	− 0.0006008
**NASH-associated cirrhosis**							
3D	GLZLM_ZP	227.2353	114.078	1.99	0.046	3.646499	450.824

*NASH* nonalcoholic steatohepatitis.

**Figure 3:**
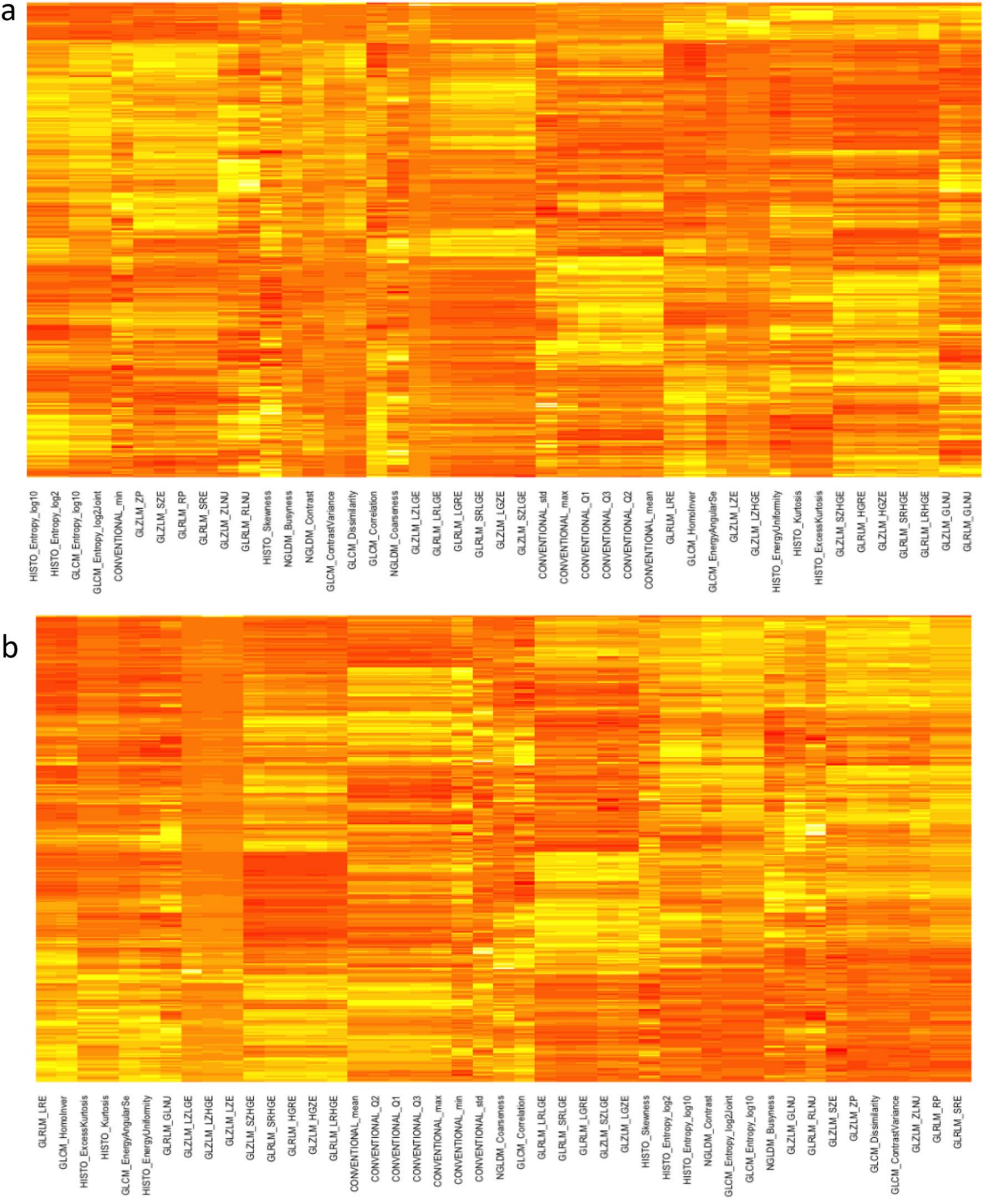
Heat maps generated from 2-dimenensional (**a**) and 3-dimensional (**b**) ROIs segmented in HBP images and demonstrating the distribution of significant features in the study population.

In least absolute shrinkage and selection operator (LASSO) analysis, the largest deviance ratio—in training (0.132 for 2D and 0.316 in 3D) and testing (0.131 for 2D and 0.196 in 3D) subsets—was observed for the model differentiating cholestatic liver disease-induced cirrhosis from noncholestatic etiologies (Table 5). The features selected with LASSO are listed in Table 6. Heat maps of the significant features were plotted (Fig. 3).

**Table 5:** Results of the least absolute shrinkage and selection operator (LASSO) logistic regression model for training and testing subsets.

	Training (n = 245)		Testing (n = 61)	
	Deviance	ratio	Deviance	ratio
**Alcoholic cirrhosis**				
2D	1.213	0.060	1.299	0.020
3D	1.179	0.094	1.178	0.086
**Cholestatic liver disease**				
2D	0.807	0.132	1.000	0.131
3D	0.669	0.316	0.798	0.196
**Viral hepatitis**				
2D	1.205	0.032	1.166	− 0.013
3D	1.073	0.072	1.552	− 0.125
**NASH cirrhosis**				
2D	0.502	0.112	0.766	0.014
3D	0.530	0.064	0.820	− 0.056

NASH, nonalcoholic steatohepatitis.

**Table 6:** Features selected using the least absolute shrinkage and selection operator (LASSO) logistic regression analysis.

	Cholestatic liver disease		Alcoholic cirrhosis		Viral hepatitis		NASH cirrhosis	
	Features selected	Deviance	Features selected	Deviance	Features selected	Deviance	Features selected	Deviance
2D	CONVENTIONAL_std	0.337	CONVENTIONAL_std	− 0.139	CONVENTIONAL_min	0.003	GLRLM_RLNU	0.196
	CONVENTIONAL_Q2	0.220	CONVENTIONAL_max	− 0.266	CONVENTIONAL_mean	0.145	GLZLM_ZLNU	0.498
	HISTO_Skewness	− 0.088	HISTO_Kurtosis	− 0.180	GLCM_Correlation	− 0.106	Constant	− 2.603
	GLCM_Dissimilarity	− 0.258	GLRLM_LRLGE	0.072	GLRLM_RLNU	− 0.084		
	GLRLM_GLNU	0.334	GLRLM_GLNU	− 0.057	Constant	− 0.789		
	GLRLM_RLNU	0.043	Constant	− 0.666				
	Constant	− 1.678						
3D	CONVENTIONAL_min	− 0.181	CONVENTIONAL_std	− 0.287	CONVENTIONAL_min	0.350	CONVENTIONAL_min	0.083
	CONVENTIONAL_std	0.608	CONVENTIONAL_Q3	− 0.254	GLZLM_LGZE	0.202	GLCM_HomoInver	− 0.546
	GLCM_HomoInver	0.610	HISTO_Kurtosis	− 0.324	GLZLM_SZLGE	0.061	GLCM_Entropy_log10	0.036
	GLRLM_RP	− 0.006	GLCM_HomoInver	− 0.038	Constant	− 1.059	NGLDM_Busyness	− 0.230
	NGLDM_Coarseness	− 0.418	GLCM_Dissimilarity	0.090			Constant	− 2.517
	GLZLM_SZE	0.778	GLRLM_RLNU	0.090				
	GLZLM_SZLGE	0.057	GLZLM_SZLGE	− 0.224				
	GLZLM_SZHGE	0.008	GLZLM_LZLGE	0.203				
	GLZLM_LZLGE	− 0.588	Constant	− 0.674				
	GLZLM_GLNU	− 0.619						
	GLZLM_ZP	− 0.760						
	Constant	− 1.803						

NASH, nonalcoholic steatohepatitis.

### Intra-class correlation coefficient (ICC)

The overall intra-class correlation coefficient (ICC) estimates and their 95% confident intervals (CI) for all features combined was 0.68 (CI 0.56–0.87) for 2D and 0.71 (CI 0.61–0.93) for 3D measurements suggesting moderate reliability^18^. Individual ICC values for the most statistically relevant features in 2D- and 3D-measurments are listed in Table 7.

**Table 7:** Individual intraclass correlation coefficient (ICC) values for the most statistically relevant features.

	ICC	
	2D ROI	3D VOI
CONVENTIONAL_std	0.754	0.441
CONVENTIONAL_Q3	0.198	0.754
HISTO_Excess Kurtosis	0.534	0.614
GLCM_Energy	0.323	0.394
GLCM_Entropy_log2	0.573	0.810
GLZLM_ZP	0.581	0.379

## Discussion

Liver cirrhosis is an increasing cause of death worldwide. Approximately 1 million people die from complications of cirrhosis all over the world each year. In addition, cirrhosis accounts for 1.6% and 2.1% of the worldwide burden of disability-adjusted life years and years of life lost, respectively^1,6^.

In the present study, we developed and evaluated a radiomics-based model to predict the etiology of liver cirrhosis from HBP images of gadoxetic acid-enhanced MRI. Gadoxetic acid-enhanced MRI has been previously investigated for the staging of liver fibrosis using radiomics analysis^10^ and a deep convolutional neural network (DCNN)^7^. To the best of our knowledge, no study to date has proposed that the etiology of liver cirrhosis can be predicted using a radiomics-based model.

The fivefold validated radiomics models created using HBP images acquired with gadoxetic acid-enhanced MRI allowed classification of the etiology of liver cirrhosis with AUCs of 0.767–0.960, accuracies of 52.8–87.6%, and positive predictive values of 0.377–0.883. The highest diagnostic accuracy of 87.6% was achieved for the 2D-based model differentiating cholestatic liver disease-induced cirrhosis from noncholestatic etiologies.

During the process of radiomics model building, we investigated different techniques, including supervised machine learning and LASSO regression analysis, to explore characteristics and identify the optimal features for model construction. LASSO turned out to several advantages as it reduces redundancy, dependency, and dimensionality of the features and thus enhances model accuracy^19^. In addition, LASSO enables the generation of interpretable models using variable selection and regularization as well as integration of selected features into a radiomics signature^19^. As for classification algorithms, we used both binary and multinomial classification. However, a binary model yields a single probability value, which can be more readily interpreted than a multinomial model, while the latter is more complex and returns multiple probability values for different etiologies of liver cirrhosis^10^. This explains the higher performance metrics we obtained for the binary model differentiating cholestatic liver disease-induced cirrhosis from noncholestatic cirrhosis.

In addition, we constructed the radiomics models using features generated from 2D ROIs and 3D VOIs. In general, segmentation of the 3D VOI was easier and faster. However, the 2D-generated models performed better as evidenced by better results in terms of accuracy, sensitivity, predictive values, and AUCs except for alcoholic cirrhosis, where the performance of the 3D-based model was better. A possible explanation might be that in 2D ROIs a more representative sample of the liver parenchyma was analyzed since an entire axial section of the liver parenchyma was segmented, while 3D VOIs only covered a volume in the right lobe, which might have rendered 3D-based models less accurate in view of the inhomogeneous distribution of parenchymal involvement as in patients with primary sclerosing cholangitis (PSC). We considered segmentation of the entire liver parenchyma for 3D VOI measurements. However, we might argue that segmentation of the whole liver in 3D VOI might be a source of bias and renders the results less accurate since it would be difficult to exclude focal lesions, large (> 5 mm) blood vessels / bile ducts, as well as regions severely affected by artefacts. Furthermore, Segmentation of the whole liver would be time consuming and our target was to investigate a gadoxetic acid-enhanced MRI-based radiomics model that could be easily integrated into routine clinical practice.

Liver biopsy is indicated if noninvasive diagnostic tests fail to yield a definitive etiology of liver cirrhosis. It is a valuable means for diagnosis and differentiation of a wide range of liver diseases such as storage and metabolic diseases, autoimmune hepatitis (AIH), fatty liver diseases, and cholestatic liver diseases (such as small-duct PSC and immunoglobulin G4-associated cholangitis)^20^. Should the radiomics model prove to be comparable to liver biopsy in identifying the etiology of liver cirrhosis, this would have important benefits for patients, since radiomics analysis does not involve any invasive procedures.

Our study has several limitations. First, we used a retrospective study design. Second, the small population size and the uneven distribution of patients among subgroups are major limitations. Only a few MRI examinations were available for cross-validation, especially for NASH- and AIH-associated cirrhosis. We had to combine patients into broader categories including multiple related etiologies—as in cholestatic liver disease and viral hepatitis-induced cirrhosis—to further improve the models. Model performance might have been better if the number of MRI examinations had been larger and more balanced across different subgroups. Future studies need to involve more patients to further explore the possibility of subgroup analysis such as identification of different etiologies of cholestatic liver disease, which is currently an indication for liver biopsy. Third, the still developing radiomics technology is another major limitation. No standardized definition of radiomics-based features has been established^21^. Fourth, the ICC had moderate reliability. This could be explained by the low number of patients, which might have hindered a robust estimation of interobserver reproducibility of the interpreted radiomics features. Fifth, we did not evaluate how model performance may be affected by various demographic characteristics and clinical settings such as patient age, aspartate transaminase-to-platelet ratio index (APRI) score, or presence of focal liver lesions. Sixth, no fixed patient characteristics for either the training or the testing collectives was feasible because of the random selection of MRI examinations in the fivefold cross-validation. Finally, we only used HBP images to evaluate radiomics features. Performance of the model might be improved by including several pulse sequences in the analysis, especially diffusion-weighted images, T1-weighted images (with and without fat suppression), and T2-weighted-images (with and without fat suppression).

In conclusion, radiomics-based analysis of hepatobiliary phase images of gadoxetic acid-enhanced MRI may be a promising noninvasive method for identifying the etiology of liver cirrhosis with better performance of the 2D- compared with the 3D-generated models. This approach needs to be validated in future prospective studies in larger patient populations.

## Patients and methods

### Patient population and study design

We retrospectively identified all patients (n = 524) with confirmed etiology of liver cirrhosis who underwent gadoxetic acid-enhanced MRI (n = 766) at our institution between January 2014 and August 2019. The etiology of liver cirrhosis was diagnosed by hepatologists primarily based on clinical examination and laboratory parameters, supported by characteristic imaging findings on gadoxetic acid-enhanced MRI such as in patients with cholestatic liver disease-induced cirrhosis. Histopathological diagnosis (i.e., liver biopsy) was reserved for patients in whom definite diagnosis of the etiology of liver cirrhosis was not confirmed by the above-mentioned methods, primarily in patients with autoimmune hepatitis-induced cirrhosis, NASH-associated cirrhosis, and patients in group 6 (other etiologies).

The study was approved by the local institutional review board (ethics committee of the Charité–Universitätsmedizin Berlin) and carried out in accordance with relevant guidelines and regulations. Informed consent was waived by the ethics committee of the Charité–Universitätsmedizin Berlin.

Inclusion criteria were: a confirmed etiology of liver cirrhosis, no previous liver transplantation or cancer-related treatment including surgical resection or locoregional interventions for liver tumors, no infiltrative or large hepatic focal lesions which could preclude segmentation of ROIs, and completion of the MRI examination. Exclusion criteria were: unconfirmed etiology of liver cirrhosis (including 35 patients (38 MRI scans) who were diagnosed with cryptogenic cirrhosis), past history of liver transplantation, liver resection or locoregional intervention for management of hepatic malignancy, presence of infiltrative or large tumor for which it was difficult to draw ROIs, and nondiagnostic image quality due to severe artifacts or technical problems during acquisition resulting in incomplete MRI examination.

**Figure 4:**
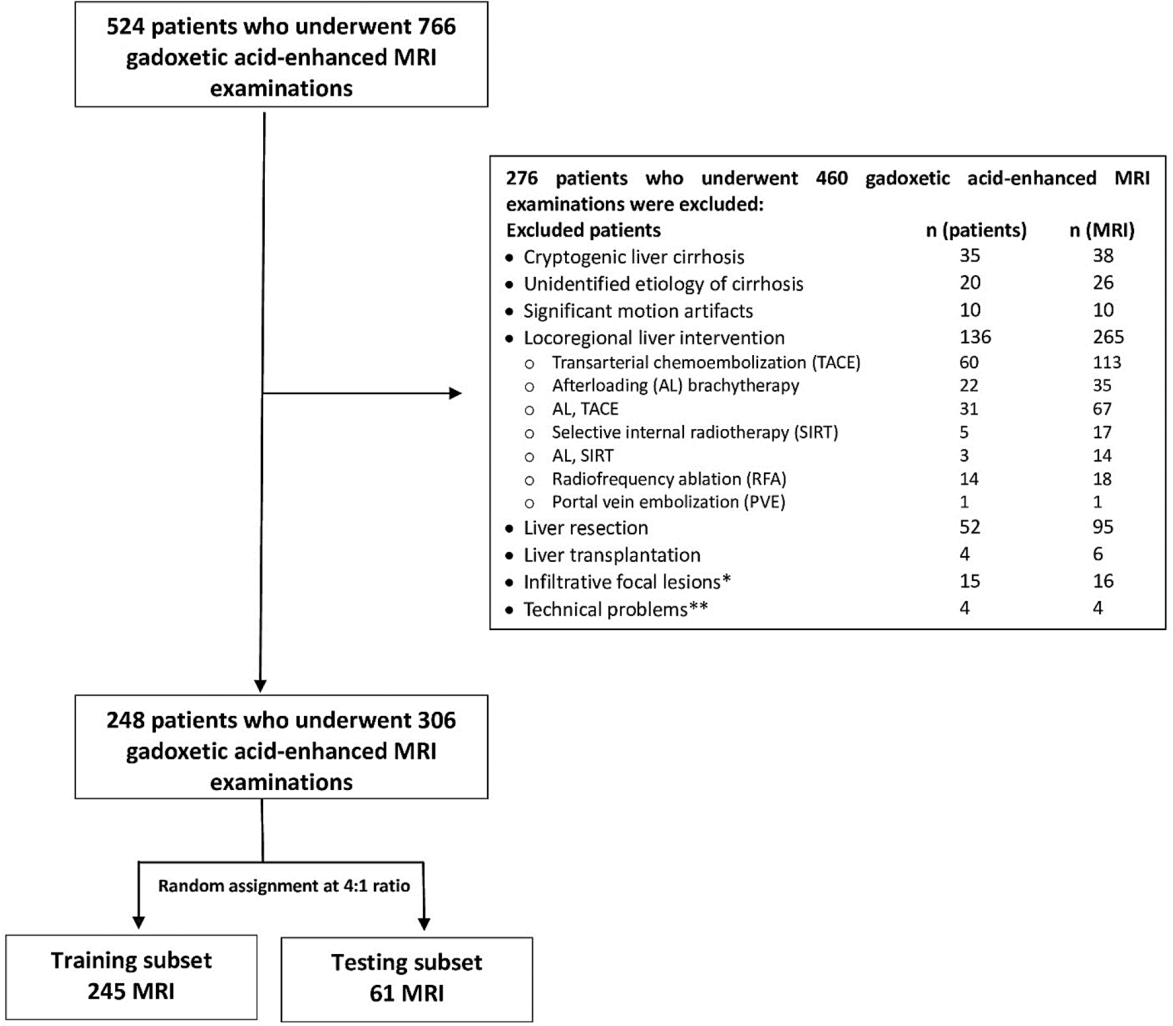
Flow chart of inclusion and exclusion of patients with liver cirrhosis who underwent gadoxetic acid-enhanced MRI. *7 patients (8 MRI) had malignant portal vein thrombosis. **MRI examinations were discontinued prematurely, and no hepatobiliary phase was acquired.

After exclusion, 248 patients who underwent 306 MRI examinations remained for analysis (Fig. 4). The study population included 8 patients (8 MRI scans) with malignant portal vein thrombosis (PVT), 4 patients (4 MRI scans) with benign PVT, and 2 patients (4 MRI scans) who were on systemic sorafenib therapy (Nexavar, Bayer Pharma AG, Berlin, Germany).

### Etiology of liver cirrhosis

MRI examinations were divided into 6 groups based on the etiology of liver cirrhosis (Table 1):

1. Group 1: Alcoholic cirrhosis (n = 108).
2. Group 2: Viral hepatitis-induced cirrhosis (n = 93).
3. Group 3: Cholestatic liver disease-induced cirrhosis (n = 58).
4. Group 4: NASH-associated cirrhosis (n = 28).
5. Group 5: AIH-associated cirrhosis (n = 8).
6. Group 6: Other etiologies (n = 11).

### Laboratory parameters and serum fibrosis/cirrhosis test

Liver function tests (aspartate aminotransferase, alanine aminotransferase, alkaline phosphatase, gamma-glutamyl transferase, serum total bilirubin, and serum albumin), kidney function tests (serum creatinine and estimated glomerular filtration rate), international normalized ratio, and platelets, performed within 1 month before or after gadoxetic acid-enhanced MRI were selected for analysis. The APRI score (n = 281) was calculated as follows: (aspartate transaminase [IU/L]/aspartate transaminase upper normal limit)/platelet count [× 10^9^/L]^22^.

### MRI examinations

All MRI examinations were performed on a 1.5 T Magnetom Aera (Siemens Healthcare, Erlangen, Germany) using an eight-channel body phased-array coil. Transverse T1-weighted images (T1WIs) (volume-interpolated breath-hold examination (VIBE) sequence covering the entire liver with 60–80 slices and an adjusted field of view of 255–300 × 340–400 mm) were acquired before and approximately 20 min after manual intravenous bolus administration of 0.1 ml per kg body weight of gadoxetic acid (Gd-EOB-DTPA, gadoxetate disodium; Primovist/Eovist, Bayer HealthCare, Berlin, Germany)^23^. Imaging parameters were as follows: repetition time (TR) of 4.58 ms, echo time (TE) of 2.25 ms, flip angle (FA) of 9°, slice thickness of 3 mm, and matrix size of 276 × 340.

### Workflow of radiomics model

The workflow included four steps: liver parenchymal segmentation, feature extraction, model construction, and, finally, model evaluation.

A reader with 10 years of experience in abdominal imaging and MRI who was blinded to the patients’ clinical and laboratory findings reviewed all MRI examinations and extracted radiomics features. To assess interobserver reproducibility using intra-class correlation coefficient (ICC), a second reader with 5 years of experience extracted the features in a randomly selected group of 30 patients. Axial VIBE T1WIs acquired approximately 20 min after gadoxetic acid administration, i.e., in the HBP, were imported into the radiomics platform as Digital Imaging and Communications in Medicine (DICOM) files. Texture analysis was performed using LIFEx software, version 5.10 (French Alternative Energies and Atomic Energy Commission, http://www.lifexsoft.org)^24^.

### Liver parenchymal segmentation

**Figure 5:**
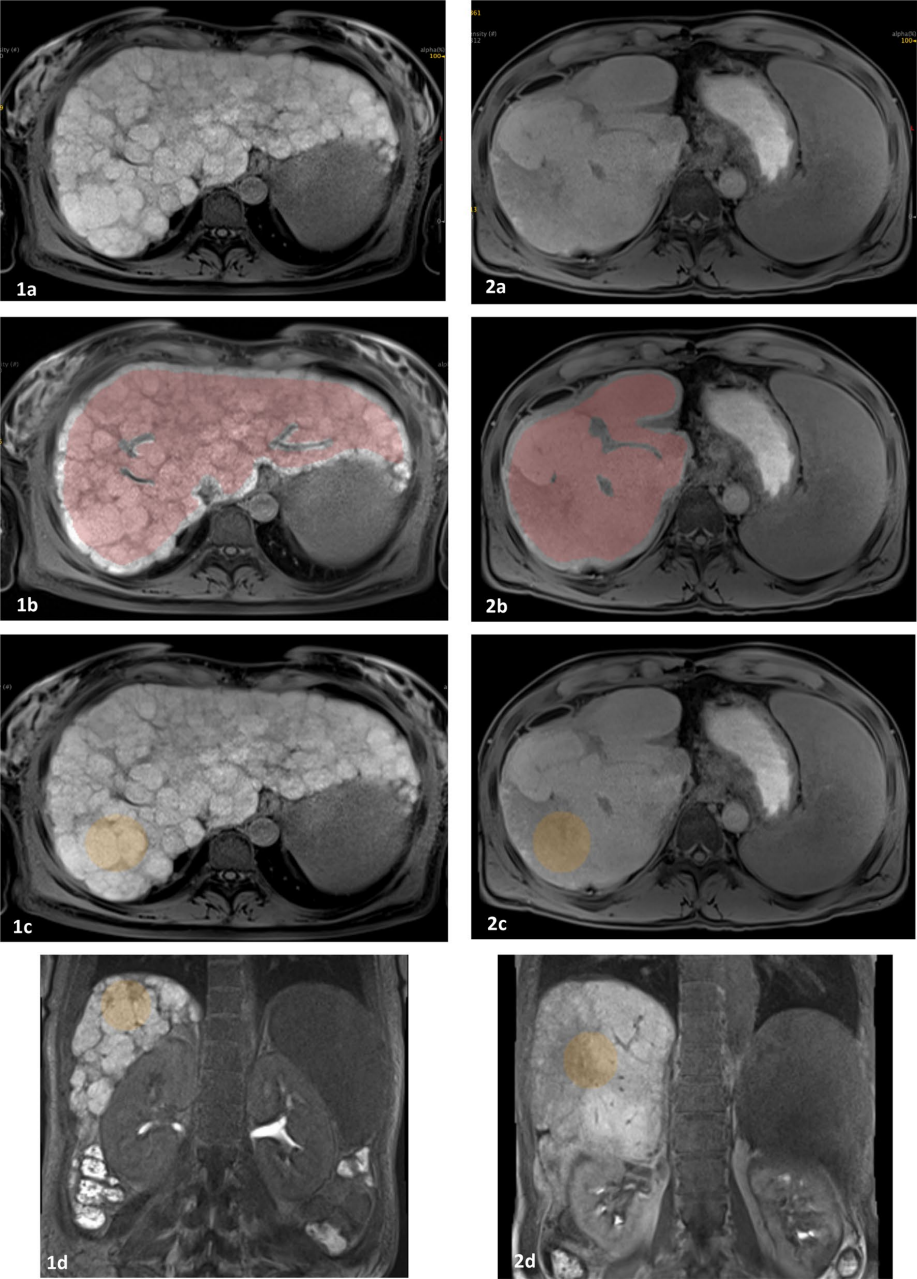
Gadoxetic acid-enhanced hepatobiliary phase (HBP) MR images showing region of interest (ROI) segmentation in two-dimensional (2D) and three-dimensional (3D) format. HBP images, axial before (**a**) and after (**b**,**c**) 2D (**b**) and 3D (**c**) ROI segmentation as well as coronal (**d**) reconstructed images showing 3D ROI segmentation. Patient 1 is a 36-year-old female with nonalcoholic steatohepatitis (NASH)-associated liver cirrhosis. Patient 2 is a 47-year-old male with primary sclerosing cholangitis complicated by liver cirrhosis.

Two-dimensional ROI and 3D VOI were segmented using drawing tools in the LIFEx software. The 2D ROI was drawn manually just above the level of the right portal vein, covering an entire slice of the liver parenchyma using 2D drawing tool (mean area, 41.56 ± 10.56 cm^2^; range, 17.8–74.3 cm^2^). A 3D VOI measuring about 40 mm^3^ (mean volume, 40.8 ± 6 cm^3^; range, 13.8–63.6 cm^3^) was segmented using 3D drawing tool, in the right posterior segment of the liver. The ROIs and VOIs were drawn 5 mm away from the liver capsule, avoiding large blood vessels (caliber ˃ 5 mm), dilated bile ducts, tumor masses, and artifacts (Fig. 5). The performance of radiomics models generated using 2D- and 3D- extracted features was compared.

### Radiomics feature extraction

A total of 45 features were extracted from the delineated ROIs. The extracted features were divided into two categories: nontextural features and textural features. In the first order, nontextural features including histogram-based indices and conventional indices were extracted. In the second or higher order, textural features were extracted based on four textural matrixes: grey-level co-occurrence matrix (GLCM), neighborhood grey-level different matrix (NGLDM), grey-level run-length matrix (GLRLM), and grey-level size zone matrix (GLSZM). The extracted features are listed in supplementary Table 1.

Preprocessing before feature extraction included image spatial resampling and gray-level normalization. Voxel sizes were resampled to the same size of 1.2 × 1.2 × 3 mm^3^ using the relative intensity resampling method between the minimum and the maximum in the VOI. Image gray-level intensity was normalized to a scale of 1 to 64^24^.

### Radiomics model construction and evaluation

All MRI examinations included in the study were randomized in a 4:1 ratio into training (n = 245) and testing (n = 61) subsets using computer-generated random numbers without matching any patient characteristics.

In the training subset, the LASSO logistic regression model with fivefold cross-validation was used to select the optimal and most informative features for predicting the etiology of liver cirrhosis. The selected features were subjected to further selection and modeling via binary logistic regression with elastic net regularization. Elimination of unreliable and statistically insignificant features was important to avoid overfitting and thus decrease running time and increase accuracy of the radiomics model^19^. A further step was to choose suitable classifiers. The radiomics signature was calculated using supervised classification algorithms. One-vs-one multiclass classification was used to differentiate between all groups (6 classes) while binary classification was used to distinguish between cholestatic and noncholestatic liver cirrhosis. The classification algorithms used are listed in supplementary Table 2. Following completion of training on classifiers, the testing subset was analyzed to determine the diagnostic performance of the models constructed in predicting the etiology of liver cirrhosis. Sensitivity, specificity, predictive values, accuracy, and receiver operating characteristic (ROC) curves were analyzed to evaluate the performance of the radiomics models.

### Statistical analysis

LASSO logistic regression was performed using Stata/MP version 16.0 (StataCorp, College Station, Texas, USA). Performance of logistic regression model was evaluated using AUC of the ROC curve. Statistical comparison of 2D and 3D ROIs was performed using the chi-square test. Classification with different methods and ROC analysis were performed with MATLAB R2019b (MathWorks, Natick, MA, USA). Heat maps of the statistically significant features were plotted using the “heatmap.2” package of R (R software version 3.6.3, R Foundation for Statistical Computing, Vienna, Austria, https://www.r-project.org). Other statistical analyses were performed with Stata/MP.

Intraclass correlation coefficient (ICC) was evaluated based on a two-way mixed-effects model for absolute agreement. The 95% confidence intervals were calculated using 1000 bootstrap iterations^25,26^. Comparison between different AUC values was calculated using the nonparametric method by DeLong et al.^27^ Categorical data are provided as absolute numbers (percentages) and continuous variables as mean ± SD. P-values < 0.05 were considered statistically significant.

## Supplementary Information

Supplementary Information.
