# Supplementary material for: A radiomics-based model to classify the etiology of liver cirrhosis using gadoxetic acid-enhanced MRI
Source: Sci Rep. 2021 May 24;11:10778. doi: 10.1038/s41598-021-90257-9 (PMC8144372; doi:10.1038/s41598-021-90257-9)
Supplement: Supplementary file 1 — Supplementary Information. [file 41598_2021_90257_MOESM1_ESM.docx]

**A radiomics-based model to classify the etiology of liver cirrhosis using gadoxetic acid-enhanced MRI**

Aboelyazid Elkilany^1,*^, Uli Fehrenbach^1^, Timo Alexander Auer^1,2^, Tobias Müller^3^, Wenzel Schöning^4^, Bernd Hamm^1^, Dominik Geisel^1^

1. Department of Diagnostic and Interventional Radiology, Charité-Universitätsmedizin Berlin, Corporate Member of Freie Universität Berlin, Humboldt-Universität zu Berlin, Berlin Institute of Health, Augustenburger Platz 1, 13353, Berlin, Germany
2. Berlin Institute of Health (BIH), Anna-Louisa-Karsch-Straße 2, 10178, Berlin, 10178, Germany
3. Division of Gastroenterology and Hepatology, Department of Medicine, Charité-Universitätsmedizin Berlin, Corporate Member of Freie Universität Berlin, Humboldt-Universität zu Berlin, Berlin Institute of Health, Augustenburger Platz 1, 13353, Berlin, Germany
4. Department of General, Visceral and Transplantation Surgery, Charité-Universitätsmedizin Berlin, Corporate Member of Freie Universität Berlin, Humboldt-Universität zu Berlin, Berlin Institute of Health, Augustenburger Platz 1, 13353, Berlin, Germany

| **Conventional indices** | **Grey-level run length matrix** | **GLRLM** |
| --- | --- | --- |
| - Conventional_min | - Short-Run Emphasis | SRE |
| - Conventional_mean | - Long-Run Emphasis | LER |
| - Conventional_std | - Low Gray-level Run Emphasis | LGRE |
| - Conventional_max | - High Gray-level Run Emphasis | HGRE |
| - Conventional_Q1 | - Short-Run Low Gray-level Emphasis | SRLGE |
| - Conventional_Q2 | - Short-Run High Gray-level Emphasis | SRHGE |
| - Conventional_Q3 | - Long-Run Low Gray-level Emphasis | LRLGE |
| **Histogram-based features** | - Long-Run High Gray-level Emphasis | LRHGE |
| - Histo_Skewness | - Gray-Level Non-Uniformity for run | GLNUr |
| - Histo_Kurtosis | - Run Length Non-Uniformity | RLNU |
| - Histo_Excess Kurtosis | - Run Percentage | RP |
| - Histo_Entropy_log10 | **Grey-level zone length matrix** | **GLZLM** |
| - Histo_Entropy_log2 | - Short-Zone Emphasis | SZE |
| - Histo_Energy Uniformierty | - Long-Zone Emphasis | LZE |
| **Grey-level co-occurrence matrix (GLCM)** | - Low Gray-level Zone Emphasis | LGZE |
| - Homogeneity | - High Gray-level Zone Emphasis | HGZE |
| - Energy | - Short-Zone Low Gray-level Emphasis | SZLGE |
| - Contrast vaiance | - Short-Zone High Gray-level Emphasis | SZHGE |
| - Correlation | - Long-Zone Low Gray-level Emphasis | LZLGE |
| - Entropy_log10 | - Long-Zone High Gray-level Emphasis | LZHGE |
| - Entropy_log2 | - Gray-Level Non-Uniformity for zone | GLNUz |
| - Dissimilarity | - Zone Length Non-Uniformity | ZLNU |
| **Neighborhood grey-level different matrix (NGLDM)** | - Zone Percentage | ZP |
| - Contrast |  |  |
| - Coarseness |  |  |
| - Busyness |  |  |

**Supplementary table 1**. List of extracted features.

| **Support vector machine (SVM)** | **Ensemble classifier** | **K-nearest neighbors (KNN)** |
| --- | --- | --- |
| Linear SVM | Boosted trees | Fine KNN |
| Quadratic SVM | Bagged | Medium KNN |
| Cubic SVM | Subspace discrimination | Coarse KNN |
| Fine Gaussian SVM | Subspace KNN | Cosine KNN |
| Medium Gaussian SVM | RUSBoost trees | Cubic KNN |
| Coarse Gaussian SVM |  | Weighted KNN |
|  |  |  |
| **Discrimination analysis** | **Decision tree** | **Naive Bayes** |
| Linear discrimination | Fine tree | Gaussian naïve Bayes |
| Quadratic discrimination | Medium tree | Kernel naïve Bayes |
|  | Coarse tree |  |
|  |  |  |
|  |  |  |
|  |  |  |
|  |  |  |

**Supplementary table 2.** List of the supervised classification algorithms used.
